# Supplementary material for: Dimerization-dependent serine protease activity of FAM111A prevents replication fork stalling at topoisomerase 1 cleavage complexes
Source: Nat Commun. 2024 Mar 7;15:2064. doi: 10.1038/s41467-024-46207-w (PMC10920703; doi:10.1038/s41467-024-46207-w)
Supplement: Supplementary file 1 — Supplementary Information [file 41467_2024_46207_MOESM1_ESM.pdf]

**a**

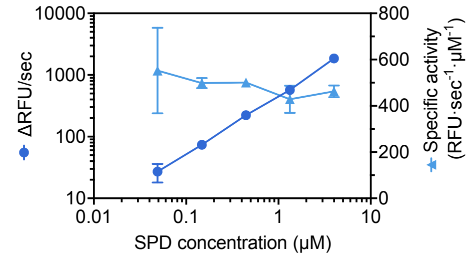


**b**

```
CLUSTAL O(1.2.4) multiple sequence alignment


Chymotrypsin      -----WQVSLQDKT-GFHF----------------CGGSLINENWVVTAAHCGVTTSDVV-       121
FAM111A           VGYLFWDSATTGYATCFVFKGLFILTCRHVIDSIVGDGIEPSKWATIIGQCVRVTFGYEE       416
                       *: :  . :  * *                *..:  .:*..  .:*  .* .

Chymotrypsin      ----------VAGEFDQGSSSEKIQKLKIAKVFKNSKYNSLTINNDITLLKLSTAASFSQ       171
FAM111A           LKDKETNYFFVEPWFEIHNEEL---DYAVLKLKENGQQVPMELYNGITPVPLSG------       467
                            *   *:  ...    .  : *: :*.:   : : *.** : **

Chymotrypsin      TVSAVCLPSASDDFAAGTTCVTTGWGLTRYTNANTPDRLQQ--ASLPLLSNTNCKKYWGT       229
FAM111A           LIHIIGHPYGEKKQI--DACAVIPQGQRA---KKCQERVQSKKAESPEYVHMYTQRSFQK       522
                   :  :  * ....     :*..   *       :  :*:*.  *. *   :   :: : .

Chymotrypsin      KIKDAMICAGASGVSSCMGDSGGPLVCKKN-------GAWTL------VGIVSWGSSTCS       276
FAM111A           IVHNPDVI--TYDTEFFFGASGSPVFDSKGSLVAMHAAGFAYTYQNETRSIIEFGSTMES       580
                   :::  :   : ...  :* **.*:. .*.       ..::        .*:.:**:  *

Chymotrypsin      TSTPGVYARVTALVNWVQQTLAAN 300
FAM111A           IL----LDIKQRHKPWYEEVFVN- 599
                                 * ::.:.
```

**Supplementary Figure 1**
(**a**) Slopes and specific activities at various enzyme concentrations in the assays shown in Fig. 1**d**. Slopes represent the change in relative fluorescence units (RFU) over time, and specific activity, representing slope values normalized by enzyme concentrations. (**b**) Amino acid sequence alignment of the human FAM111A SPD and bovine chymotrypsin generated by Clustal Omega. Asterisks (*): identical residue; colons (:): conserved substitution; period (.): semi-conserved substitution. Catalytic residues are indicated in red. Source data are provided as a Source Data file.

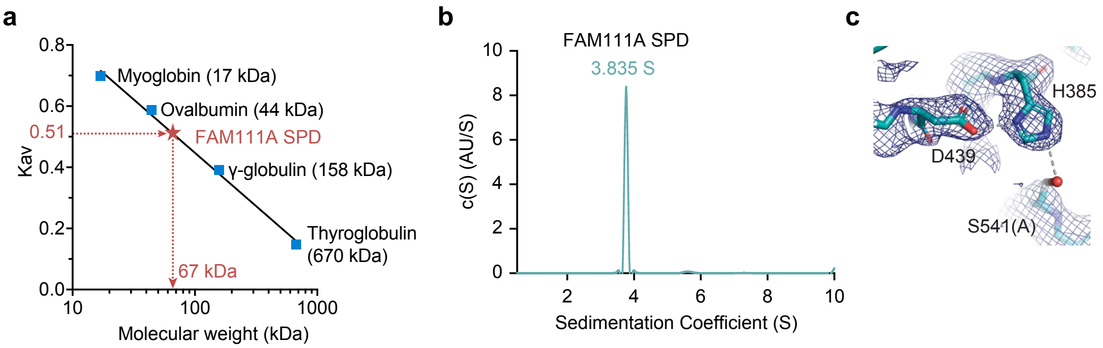


**Supplementary Figure 2**

(**a**) Calibration curve for the standard proteins on the Superdex 200 10/300 column. Kav values of the indicated standard proteins were plotted against their known molecular masses to generate a standard curve for molecular mass. The molecular mass of FAM111A SPD protein was estimated to be 67 kDa based on its Kav value of 0.51. Kav is defined as the ratio of the difference between the elution volume (Ve) and the void volume (Vo) to the difference between the total bed volume (Vt) and the void volume: Kav = (Ve - Vo)/(Vt - Vo). (**b**) Analytical ultracentrifugation analysis demonstrates that purified FAM111A SPD is a dimer. The graph represents the sedimentation coefficient distribution c(s) profile attained by sedimentation velocity (SV) data analysis. (**c**) Electron density map of the SPD active site. A 2mFo-dFc electron density map contoured at 1σ showing density for the catalytic triad of the SPD. A serine residue at position 541 is shown modelled (gray) along with the predicted hydrogen bond to H385. Source data are provided as a Source Data file.

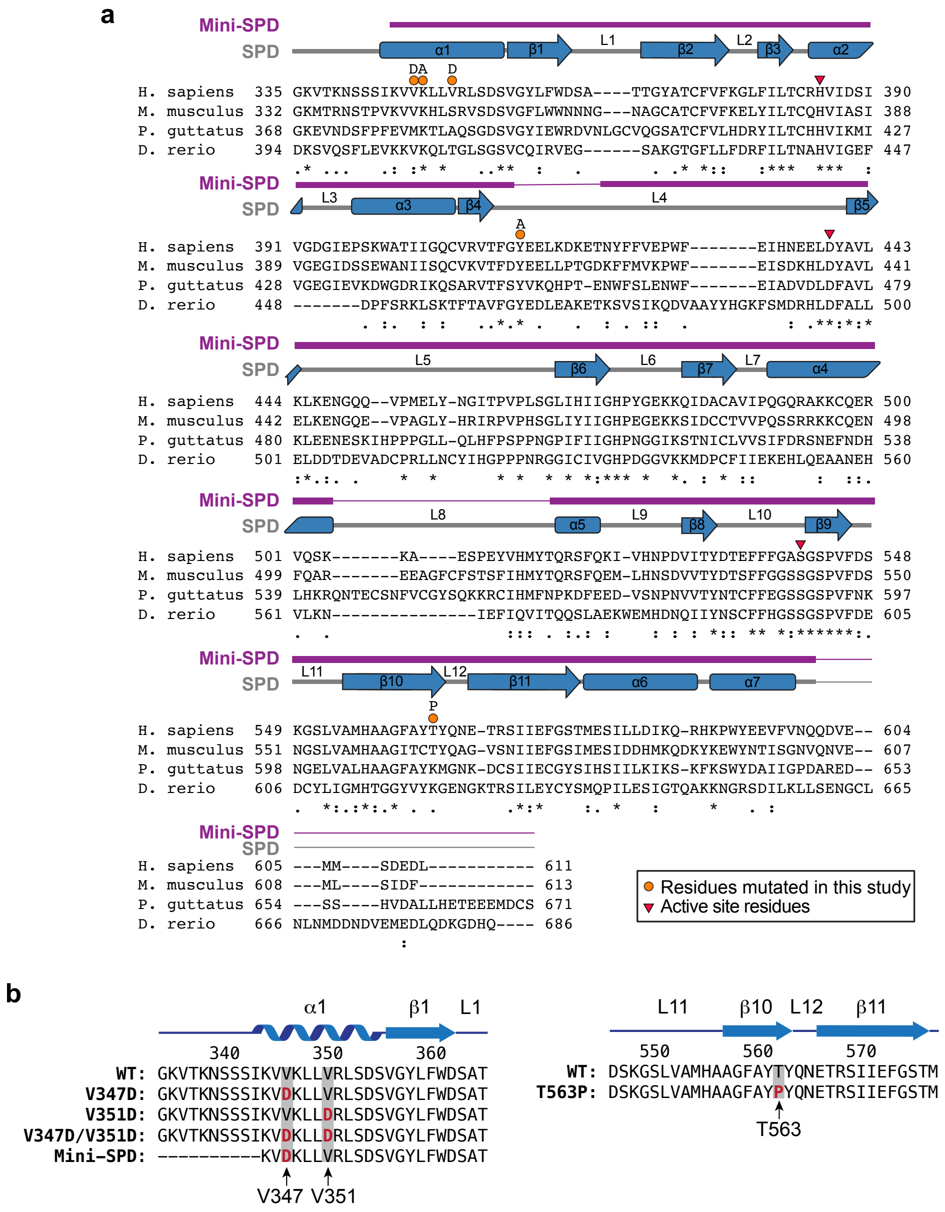


**Supplementary Figure 3**

(**a**) Multiple sequence alignment (MSA) with *Homo sapiens* FAM111A, *Mus musculus* FAM111A, *Patherophis guttatus* FAM111A, and *Danio rerio* FAM111A-like SPDs were generated using Clustal Omega. Alignment consensus symbols from Clustal Omega are indicated. Asterisks (*): identical residue; colons (:): conserved substitution; period (.): semi-conserved substitution. The gray lines indicate the region included in the constructs of *H. sapiens* FAM111A SPD, and secondary structures determined in this study are annotated in blue. Purple lines indicate the region included in the mini-SPD construct. In both SPD (gray) and mini-SPD (purple), the thick and thin lines indicate the regions observed and not observed in the structures, respectively. Conserved catalytic triad residues are marked in red arrowheads. Orange dots indicate residues mutated in this study. (**b**) A sequence alignment of the α1 helices and β10 strand in various SPD proteins. The positions of V347, V351, and T563 are highlighted in gray, and introduced mutations are indicated in red.

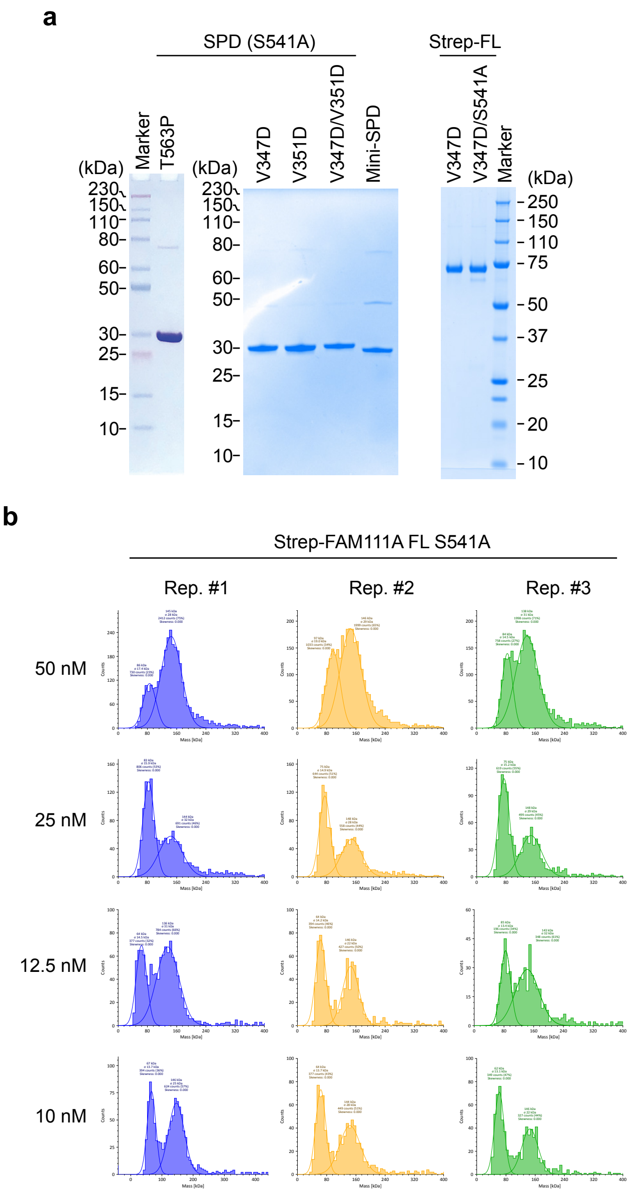


**Supplementary Figure 4**

(**a**) Purified recombinant proteins with various mutations analyzed by SDS-PAGE. The gel was stained with Coomassie Brilliant Blue. Positions of molecular weight marker proteins are indicated. (**b**) Mass distribution for Strep-FAM111A S541A obtained by mass photometry. Measurements were taken at indicated concentrations in three technical replicates (Rep. #1-3). Source data are provided as a Source Data file.

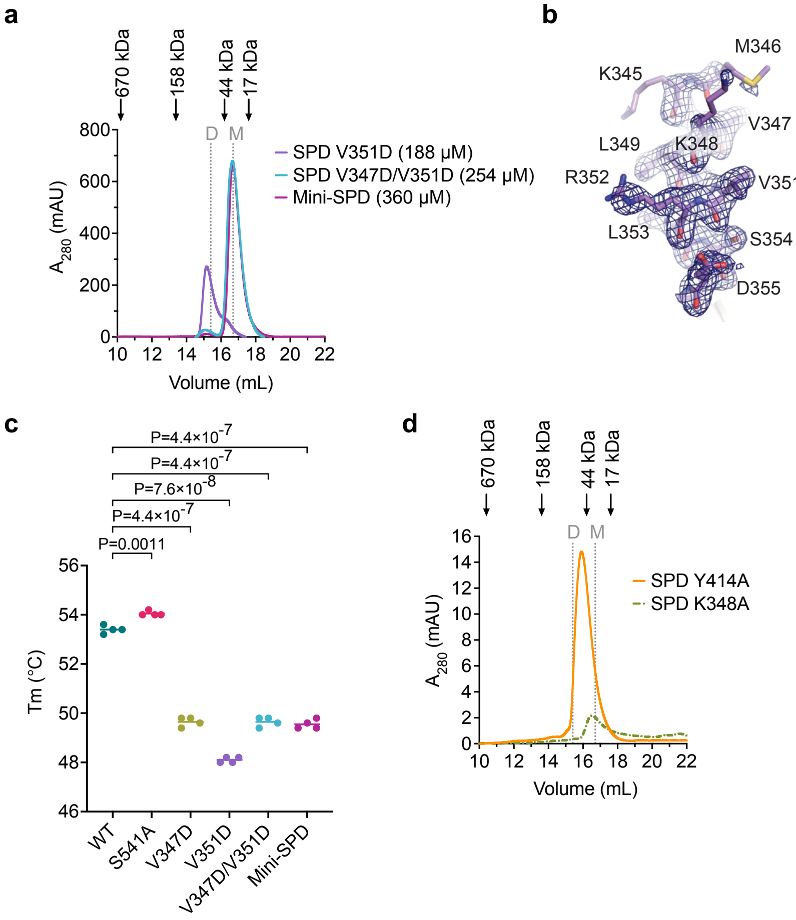


| SPD | Tm (°C) |
|---|---|
| WT | 53.4 ± 0.2 |
| S541A | 54.1 ± 0.2 |
| V347D | 49.7 ± 0.2 |
| V351D | 48.1 ± 0.2 |
| V347D/V351D | 49.7 ± 0.2 |
| Mini-SPD | 49.6 ± 0.2 |

**Supplementary Figure 5**

(**a**) Size-exclusion chromatogram of high concentration monomeric mutants on Superdex 200 Increase 10/300 GL. Absorbance at 280 nm (A280) is shown. Concentrations of injected proteins are shown in the legends. Elution volumes for SEC standard proteins are indicated with arrows on top. Expected theoretical elution volumes for an SPD monomer (31.5 kDa, indicated with a letter M) and an SPD dimer (63.0 kDa, indicated with a letter D) are shown with dotted lines. (**b**) Electron density map of helix α1 of mini-SPD. A 2mFo-dFc electron density map contoured at 1σ shows density for helix α1 backbone and most sidechains are visible. (**c**) Thermal stability of purified SPD and monomeric mutant proteins. The melting temperatures (Tm) of the indicated proteins were measured by thermal shift assays. Top: a graph showing the Tm values of individual measurements (line: mean). Significance of differences between mutants and WT was determined by two-tailed unpaired t-test. Bottom: Mean ± error of four replicates. Error is the higher value of the Instrument Limit of Error (ILE)=0.2°C and s.d. (**d**) Size-exclusion chromatogram of the Y414A and K348A mutants. Absorbance at 280 nm (A280) is shown. Elution volumes for SEC standard proteins are indicated with arrows on top. Expected theoretical elution volumes for an SPD monomer (31.5 kDa, indicated with a letter M) and an SPD dimer (63.0 kDa, indicated with a letter D) are shown with dotted lines. Source data are provided as a Source Data file.

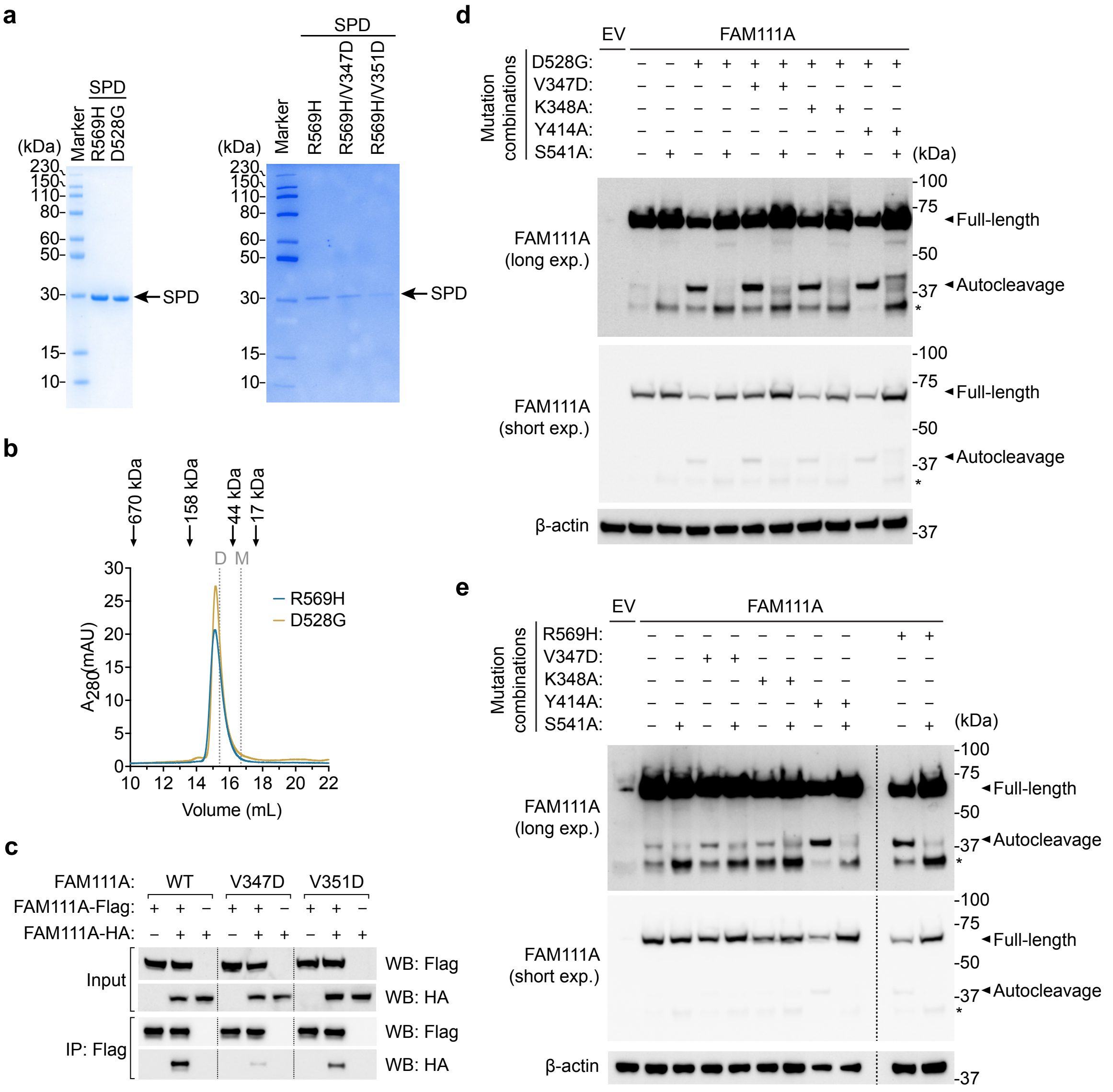


**Supplementary Figure 6**

(**a**) SDS-PAGE analysis of purified recombinant SPD proteins. The gel was stained with Coomassie Brilliant Blue. Positions of molecular weight marker proteins are indicated. (**b**) Size-exclusion chromatogram of the SPD R569H and D528G mutants. Absorbance at 280 nm (A280) is shown. Elution volumes for SEC standard proteins are indicated with arrows on top. Expected theoretical elution volumes for an SPD monomer (31.5 kDa, indicated with a letter M) and an SPD dimer (63.0 kDa, indicated with a letter D) are shown with dotted lines. (**c**) Intermolecular self-interactions of FAM111A (WT, V347D, or V351D) assessed by co-immunoprecipitation. FAM111A proteins with either a Flag or an HA tag were coexpressed in 293T cells. Inputs (top) and anti-Flag precipitation samples (bottom) are analyzed by Western blotting (WB) using indicated antibodies. (**d,e**) FAM111A autocleavage. FAM111A proteins with indicated combinations of mutations were transiently expressed in 293T cells (which lacks endogenous FAM111A expression) and examined by Western blotting. Long (top) and short (middle) exposures of anti-FAM111A blots are shown. β-actin (bottom) is shown as a loading control. The positions of full-length and autocleavage bands are indicated by black arrowheads. *Nonspecific degradation bands. Source data are provided as a Source Data file.

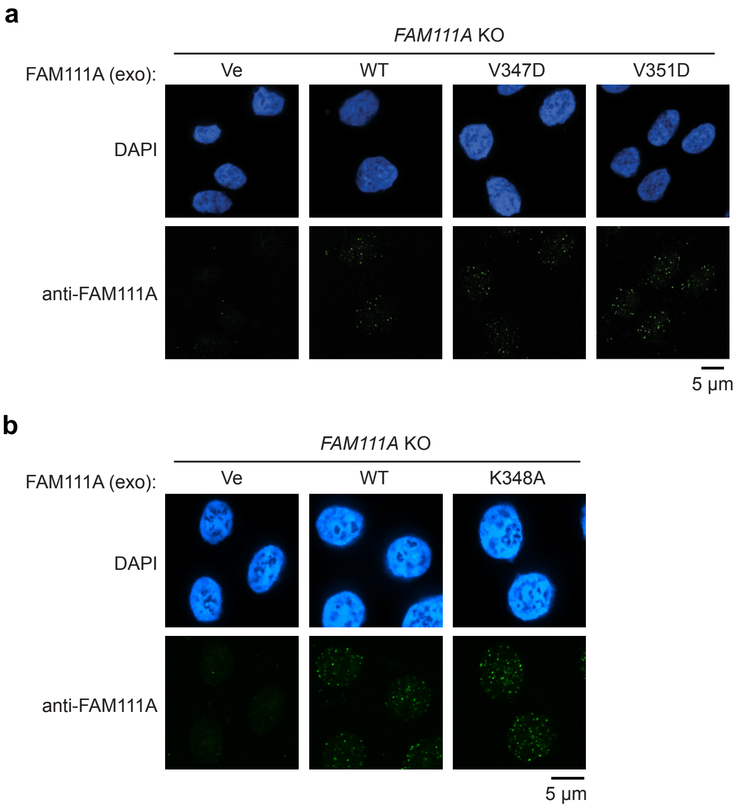


**Supplementary Figure 7**
(**a**,**b**) Immunofluorescence microscope images of FAM111A foci on chromatin. The indicated FAM111A proteins were stably expressed exogenously (exo) by lentiviral vectors in FAM111A KO HAP1 cells. Cells were pre-extracted with 0.5% TX-100 before fixation with methanol:acetone (3:1) and costaining with DAPI (blue) and anti-FAM111A antibody (green, pseudo-colored from orange). Ve: empty vector. Images are single z slices. Scale bar, 5 μm.

| P1 residue: | FAM111A (20 μM) Activity | FAM111A Rate Relative to P1=F | Chymotrypsin (1.7 pM) Activity | Chymotrypsin Rate Relative to P1=F |
|---|---|---|---|---|
| R | -1.9 ±1.3 | 0 | | |
| V | 0.0 ±0.2 | 0 | | |
| F | 3990 ±30 | 1 | 5700 ±500 | 1 |
| Y | 2.6 ±0.5 | $6.5 \times 10^{-4}$ | 90 ±10 | $1.6 \times 10^{-2}$ |
| W | 10.2 ±0.9 | $2.6 \times 10^{-3}$ | 190 ±30 | $3.3 \times 10^{-2}$ |

**Supplementary Table 1. Protease Specificity for the P1 Residue**. Chymotrypsin or FAM111A were incubated with AMC-peptide substrates with the indicated residue in the P1 position and protease activity was monitored as an increase in AMC fluorescence. The rate from reactions containing catalytically inactive (S541A) SPD were used to correct for background in FAM111A assays, and buffer-only reactions were used to correct for background for reactions containing chymotrypsin. Substrate specificity defined as the rate relative to phenylalanine are shown. N=4, error bars ±s.d. Experiments were repeated twice and similar results were obtained.

**Supplementary Table 2.** X-ray crystallographic data collection and refinement statistics

| Structure | SPD S541A | Mini SPD |
|---|---|---|
| PDB Entry ID: | 8S9K | 8S9L |
| **Data collection** | | |
| Space group | $P\ 2_12_12_1$ | $P\ 2_12_12_1$ |
| Cell dimensions | | |
| *a*, *b*, *c* (Å) | 74.81, 83.76, 191.12 | 68.46,70.03,128.02 |
| $\alpha, \beta, \gamma$ (°) | 90, 90, 90 | 90, 90, 90 |
| Resolution (Å) | 50-2.7 (2.8-2.7) | 70-1.85 (1.92-1.85) |
| $R_{sym}$ or $R_{merge}$ | 0.026 (1.37) | 0.061 (1.79) |
| $I / \sigma I$ | 8.3 (1.4) | 32.2 (1.3) |
| Completeness (%) | 99.8 (100) | 99.8 (100) |
| Redundancy | 6.8 (7.1) | 6.6 (6.9) |
| | | |
| **Refinement** | | |
| Resolution (Å) | 50-2.7 | 50-1.85 |
| No. reflections | 32707 | 53161 |
| $R_{work}$ / $R_{free}$ | 0.196/ 0.249 | 0.218/0.252 |
| No. atoms | | |
| Protein | 8279 | 3799 |
| Ligand/ion | 6 | 5 |
| Water | 21 | 90 |
| *B*-factors (Å$^2$) | | |
| Protein | 65.2 | 67.8 |
| Ligand/ion | 60.9 | 134 |
| Water | 50.8 | 56.4 |
| R.m.s. deviations | | |
| Bond lengths (Å) | 0.002 | 0.012 |
| Bond angles (°) | 0.52 | 1.10 |

*Values in parentheses are for the highest-resolution shell (10% of reflections).
