# Supplementary material for: Dimerization-dependent serine protease activity of FAM111A prevents replication fork stalling at topoisomerase 1 cleavage complexes
Source: Nat Commun. 2024 Mar 7;15:2064. doi: 10.1038/s41467-024-46207-w (PMC10920703; doi:10.1038/s41467-024-46207-w)
Supplement: Supplementary file 3 — Reporting Summary [file 41467_2024_46207_MOESM3_ESM.pdf]

nature portfolio

Corresponding author(s): Matthew J. Schellenberg and Yuichi J. Machida

Last updated by author(s): Feb 3, 2024

Reporting Summary

Nature Portfolio wishes to improve the reproducibility of the work that we publish. This form provides structure for consistency and transparency in reporting. For further information on Nature Portfolio policies, see our Editorial Policies and the Editorial Policy Checklist.

Statistics

For all statistical analyses, confirm that the following items are present in the figure legend, table legend, main text, or Methods section.

n/a	Confirmed	
☐	☒	The exact sample size (*n*) for each experimental group/condition, given as a discrete number and unit of measurement
☐	☒	A statement on whether measurements were taken from distinct samples or whether the same sample was measured repeatedly
☐	☒	The statistical test(s) used AND whether they are one- or two-sided<br>*Only common tests should be described solely by name; describe more complex techniques in the Methods section.*
☒	☐	A description of all covariates tested
☐	☒	A description of any assumptions or corrections, such as tests of normality and adjustment for multiple comparisons
☐	☒	A full description of the statistical parameters including central tendency (e.g. means) or other basic estimates (e.g. regression coefficient) AND variation (e.g. standard deviation) or associated estimates of uncertainty (e.g. confidence intervals)
☐	☒	For null hypothesis testing, the test statistic (e.g. *F*, *t*, *r*) with confidence intervals, effect sizes, degrees of freedom and *P* value noted<br>*Give P values as exact values whenever suitable.*
☒	☐	For Bayesian analysis, information on the choice of priors and Markov chain Monte Carlo settings
☒	☐	For hierarchical and complex designs, identification of the appropriate level for tests and full reporting of outcomes
☒	☐	Estimates of effect sizes (e.g. Cohen's *d*, Pearson's *r*), indicating how they were calculated

Our web collection on statistics for biologists contains articles on many of the points above.

Software and code

Policy information about availability of computer code

Data collection	NIS Elements<br>Unicorn (version 5.11 and 7.3)<br>Clariostar (version 5.61)<br>SoftMax Pro (version 7.0.3)<br>Acquire MP (2023 R1)<br>CFX Manager (version 3.1)
Data analysis	Image Lab (version 6.0.1)<br>GraphPad Prism (version 9.5.0)<br>R (version 4.1.2)<br>Fiji (version 2.9.0)<br>HKL2000 (version 720)<br>Coot (version 0.8.9.1)<br>Phenix (version 1.17.1-3660)<br>PyMOL (version 2.5.2)<br>ChimeraX (version 1.6.1)<br>Rosetta (version 2019.3-11)<br>GROMACS (version 2021.4)<br>FiberStudio (version 3.2.7)

MARS (version 3.41)
Discover MP (2023 R1)
Sedfit (version 15.01b)
Clustal Omega (version 1.2.4)

For manuscripts utilizing custom algorithms or software that are central to the research but not yet described in published literature, software must be made available to editors and reviewers. We strongly encourage code deposition in a community repository (e.g. GitHub). See the Nature Portfolio guidelines for submitting code & software for further information.

Data

Policy information about availability of data

All manuscripts must include a data availability statement. This statement should provide the following information, where applicable:

- Accession codes, unique identifiers, or web links for publicly available datasets
- A description of any restrictions on data availability
- For clinical datasets or third party data, please ensure that the statement adheres to our policy

Atomic coordinates and structure factors have been deposited in the Protein Data Bank (PDB) under accession numbers 8S9K (SPD S541A) [http://doi.org/10.2210/pdb8S9K/pdb] and 8S9L (mini-SPD) [http://doi.org/10.2210/pdb8S9L/pdb]. Previously published protein structure data used for the analysis in this study is available in the Protein Data Bank (www.rcsb.org) under PDB ID: 7GCH (chymotrypsin co-crystallized with a TFMK transition state analog). The source data file, containing uncropped images of gels/blots and raw data used for generating graphs, is provided with this paper. The configuration files for the MD simulations have been uploaded in Figshare.

Research involving human participants, their data, or biological material

Policy information about studies with human participants or human data. See also policy information about sex, gender (identity/presentation), and sexual orientation and race, ethnicity and racism.

Reporting on sex and gender	N/A
Reporting on race, ethnicity, or other socially relevant groupings	N/A
Population characteristics	N/A
Recruitment	N/A
Ethics oversight	N/A

Note that full information on the approval of the study protocol must also be provided in the manuscript.

Field-specific reporting

Please select the one below that is the best fit for your research. If you are not sure, read the appropriate sections before making your selection.

[x] Life sciences [ ] Behavioural & social sciences [ ] Ecological, evolutionary & environmental sciences

For a reference copy of the document with all sections, see nature.com/documents/nr-reporting-summary-flat.pdf

Life sciences study design

All studies must disclose on these points even when the disclosure is negative.

Sample size	No sample size calculation was performed to pre-determine sample sizes. A minimum sample size of 3 replicates was chosen for protease assays to provide an adequate estimate of error associated with each data point in an assay. When adequate reagents were available a fourth replicate was included. Sample sizes for focus formation (Fig. 7c-f) and DNA combing assays (Fig. 7h,i) were determined based on the previous study (Kojima et al., https://doi.org/10.1038/s41467-020-15170-7), which showed the minimum of n=100 was sufficient to reliably determine the effect of FAM111A knockout.
Data exclusions	A pre-determined criterion was applied in enzyme assays to exclude data showing obvious deviations from other technical replicates, or inconsistent readings that precluded slope measurement needed to calculate rates, which is often caused by air bubbles in assay wells.
Replication	Experiments were repeated at least twice and similar results were obtained. Number of replication for each experiment was described in figure legends.
Randomization	For the drug treatment experiments in Fig. 7h,i, cells were randomly allocated to each treatment. Randomization was not used for other experiments as no grouping was performed.
Blinding	The investigator was blinded to sample identity for focus formation (Fig. 7c-f) and DNA combing assays (Fig. 7h,i). Blinding was not relevant to other experiments as no measure was subjective.

Reporting for specific materials, systems and methods

We require information from authors about some types of materials, experimental systems and methods used in many studies. Here, indicate whether each material, system or method listed is relevant to your study. If you are not sure if a list item applies to your research, read the appropriate section before selecting a response.

Materials & experimental systems

n/a	Involved in the study
☐	☒ Antibodies
☐	☒ Eukaryotic cell lines
☒	☐ Palaeontology and archaeology
☒	☐ Animals and other organisms
☒	☐ Clinical data
☒	☐ Dual use research of concern
☒	☐ Plants

Methods

n/a	Involved in the study
☒	☐ ChIP-seq
☒	☐ Flow cytometry
☒	☐ MRI-based neuroimaging

Antibodies

Antibodies used

1. Rabbit anti-FAM111A (Abcam, ab184572, 1:1,000 for WB, 1:200 for IF)
2. Mouse anti-β-actin [clone AC-74] (Sigma, A5316, 1:5,000 for WB)
3. Rabbit anti-Flag (Cell Signaling, #2368, 1:1,000 for WB)
4. Rabbit anti-HA (Santa Cruz, sc-805, 1:1,000 for WB)
5. Mouse anti-TOP1cc (a gift from Scott Kaufmann, 1:200 for IF)
6. Goat anti-rabbit IgG Alexa Fluor 568 (Invitrogen, A-11036, 1:2,000 for IF)
7. Goat anti-mouse IgG Alexa Fluor 488 (Invitrogen, A-11029, 1:1,000 for IF)
8. Rat anti-CldU [clone BU1/75 (ICR1)] (Abcam, ab6326, 1:100 for DNA combing)
9. Mouse anti-IdU [clone B44] (BD, 347580, 1:20 for DNA combing)
10. Mouse anti-single strand DNA [clone 16-19] (Millipore, MAB3034, 1:200 for DNA combing)
11. Cy5-labeled goat anti-rat IgG (Abcam, ab6565, 1:100 for DNA combing)
12. Cy3-labeled goat anti-mouse IgG (Abcam, ab97035, 1:100 for DNA combing)

Validation

Antibodies were validated by suppliers, previous publications, or in this study as indicated below.
1. Rabbit anti-FAM111A: Validated by FAM111A knockout cell lines (Kojima et al., https://doi.org/10.1038/s41467-020-15170-7)
2. Mouse anti-β-actin: https://www.sigmaaldrich.com/US/en/product/sigma/a5316
3. Rabbit anti-Flag: Validated by expression of FAM111A-Flag (Supplementary Fig. 6c)
4. Rabbit anti-HA: Validated by expression of FAM111A-HA (Supplementary Fig. 6c)
5. Mouse anti-TOP1cc: Validated for immunofluorecence using topoisomerase inhibitors and a topo I-deficient cell line (Patel et a., https://doi.org/10.1093/nar/gkw109)
6. Goat anti-rabbit IgG Alexa Fluor 568: https://www.thermofisher.com/antibody/product/Goat-anti-Rabbit-IgG-H-L-Highly-Cross-Adsorbed-Secondary-Antibody-Polyclonal/A-11036
7. Goat anti-mouse IgG Alexa Fluor 488: https://www.thermofisher.com/antibody/product/Goat-anti-Mouse-IgG-H-L-Highly-Cross-Adsorbed-Secondary-Antibody-Polyclonal/A-11029
8. Rat anti-CldU: https://www.abcam.com/products/primary-antibodies/brdu-antibody-bu175-icr1-proliferation-marker-ab6326.html
9. Mouse anti-IdU: https://www.bdbiosciences.com/en-us/products/reagents/flow-cytometry-reagents/clinical-discovery-research/single-color-antibodies-ruo-gmp/purified-mouse-anti-brdu.347580
10. Mouse anti-single strand DNA: https://www.emdmillipore.com/US/en/product/Anti-DNA-Antibody-single-stranded-clone-16-19,MM_NF-MAB3034
11. Cy5-labeled goat anti-rat IgG: https://www.abcam.com/products/secondary-antibodies/goat-rat-igg-hl-cy5--preadsorbed-ab6565.html
12. Cy3-labeled goat anti-mouse IgG: https://www.abcam.com/products/secondary-antibodies/goat-mouse-igg-hl-cy3--preadsorbed-ab97035.html

Eukaryotic cell lines

Policy information about cell lines and Sex and Gender in Research

Cell line source(s)

HAP1 (Horizon Discovery, #C631)
HAP1 FAM111A KO #14 (Kojima et al., https://doi.org/10.1038/s41467-020-15170-7)
293T (ATCC, #CRL-11268)

Authentication

Cell lines used in this study were not authenticated by our group.

Mycoplasma contamination

Cell lines were used from the frozen stocks that were tested negative for mycoplasma contamination using LookOut Mycoplasma PCR detection kit (Sigma) on the dates listed below.
HAP1 (07/27/2020)

	HAP1 FAM111A KO #14 (07/27/2020)<br>293T (10/25/2019)
Commonly misidentified lines<br>(See ICLAC register)	No commonly misidentified cell lines were used in this study.

Plants

Seed stocks	N/A
Novel plant genotypes	N/A
Authentication	N/A
